# Effect of the Bioprotective Properties of Lactic Acid Bacteria Strains on Quality and Safety of Feta Cheese Stored under Different Conditions

**DOI:** 10.3390/microorganisms12091870

**Published:** 2024-09-10

**Authors:** Angeliki Doukaki, Olga S. Papadopoulou, Antonia Baraki, Marina Siapka, Ioannis Ntalakas, Ioannis Tzoumkas, Konstantinos Papadimitriou, Chrysoula Tassou, Panagiotis Skandamis, George-John Nychas, Nikos Chorianopoulos

**Affiliations:** 1 Laboratory of Microbiology and Biotechnology of Foods, Department of Food Science and Human Nutrition, School of Food and Nutritional Sciences, Agricultural University of Athens, Iera Odos 75, 11855 Athens, Greece; angieduk@gmail.com (A.D.); antwniamb@gmail.com (A.B.); siapkamarina8@gmail.com (M.S.); intalakas98@gmail.com (I.N.); itzoumkas@gmail.com (I.T.); gjn@aua.gr (G.-J.N.); 2 Institute of Technology of Agricultural Products, Hellenic Agricultural Organization—DIMITRA, S. Venizelou 1, 14123 Lycovrissi, Greece; olgapapadopoulou@elgo.gr (O.S.P.); ctassou@elgo.gr (C.T.); 3 Laboratory of Food Quality Control and Hygiene, Department of Food Science and Human Nutrition, School of Food and Nutritional Sciences, Agricultural University of Athens, Iera Odos 75, 11855 Athens, Greece; kpapadimitriou@aua.gr (K.P.); pskan@aua.gr (P.S.)

**Keywords:** white brined cheese, aerobic packaging, edible films, *Listeria monocytogenes*, Fourier transform infrared (FTIR) spectroscopy, multispectral imaging (MSI) analysis

## Abstract

Lately, the inclusion of additional lactic acid bacteria (LAB) strains to cheeses is becoming more popular since they can affect cheese’s nutritional, technological, and sensory properties, as well as increase the product’s safety. This work studied the effect of *Lactiplantibacillus pentosus* L33 and *Lactiplantibacillus plantarum* L125 free cells and supernatants on feta cheese quality and *Listeria monocytogenes* fate. In addition, rapid and non-invasive techniques such as Fourier transform infrared (FTIR) and multispectral imaging (MSI) analysis were used to classify the cheese samples based on their sensory attributes. Slices of feta cheese were contaminated with 3 log CFU/g of *L. monocytogenes*, and then the cheese slices were sprayed with (i) free cells of the two strains of the lactic acid bacteria (LAB) in co-culture (F, ~5 log CFU/g), (ii) supernatant of the LAB co-culture (S) and control (C, UHT milk) or wrapped with Na-alginate edible films containing the pellet (cells, FF) or the supernatant (SF) of the LAB strains. Subsequently, samples were stored in air, in brine, or in vacuum at 4 and 10 °C. During storage, microbiological counts, pH, and water activity (a_w_) were monitored while sensory assessment was conducted. Also, in every sampling point, spectral data were acquired by means of FTIR and MSI techniques. Results showed that the initial microbial population of Feta was ca. 7.6 log CFU/g and consisted of LAB (>7 log CFU/g) and yeast molds in lower levels, while no *Enterobacteriaceae* were detected. During aerobic, brine, and vacuum storage for both temperatures, pathogen population was slightly postponed for S and F samples and reached lower levels compared to the C ones. The yeast mold population was slightly delayed in brine and vacuum packaging. For aerobic storage at 4 °C, an elongation in the shelf life of F samples by 4 days was observed compared to C and S samples. At 10 °C, the shelf life of both F and S samples was extended by 13 days compared to C samples. FTIR and MSI analyses provided reliable estimations of feta quality using the PLS-DA method, with total accuracy (%) ranging from 65.26 to 84.31 and 60.43 to 89.12, respectively. In conclusion, the application of bioprotective LAB strains can result in the extension of feta’s shelf life and provide a mild antimicrobial action against *L. monocytogenes* and spoilage microbiota. Furthermore, the findings of this study validate the effectiveness of FTIR and MSI techniques, in tandem with data analytics, for the rapid assessment of the quality of feta samples.

## 1. Introduction

Fermented foods have gained consumer attention in recent years, with dairy products prominently featured. Feta is a Greek Protected Designation of Origin (PDO) cheese, according to Regulation (EC) No. 1829/2002 of the Commission [1], and is among the most recognized traditional Greek cheeses in the world, while it is part of the daily diet of Greeks. Feta is a traditional white-brined soft cheese that is made exclusively from sheep’s milk, or with the addition of goat’s milk in a maximum percentage of 30% [1]. Greece is a world leader in the production of sheep’s milk and ranks second in the EU for goat’s milk [2]. Feta is produced by small and medium enterprises (SMEs), as well as by large dairy industries, and is considered as an important economic activity. The goal is to meet the needs of the national market and export requirements since feta is considered a prominent Greek product.

Feta cheese is mainly produced using commercial starter cultures (mesophilic or mixtures of mesophilic and thermophilic LAB strains like *Lactococcus lactis* subsp. *Lactis*, subsp. *Cremoris*, and/or *Lb. bulgaricus*) to standardize the fermentation process due to the importance of rapid acidification for the quality of the cheese [3,4,5]. Previous studies on the microbiological quality of Feta during manufacture, ripening, and storage have shown that the low pH (4.4–4.6) and the high salt content of the curd (about 3%) during ripening reduce the viability of starter populations [5,6,7]. Conversely, these conditions seem to favor the growth of non-starter LAB, including *Lactiplantibacillus plantarum*, *Lacticaseibacillus paracasei* subsp. *paracasei*, *Levilactobacillus brevis*, *Lentilactobacillus hilgardii*, *Loigolactobacillus coryniformis*, and *Limosilactobacillus fermentum*, which have been found during the storage of ready-to-eat cheese [8,9,10]. The microbial diversity of feta is influenced by the addition of commercial starter cultures, the methods used in dairy farms, and the ripening period [11,12]. Additionally, the dairy farm environment contributes to the native milk microbiota, which ultimately affects the cheese’s microbiota [8,13]. Recent scientific articles have thoroughly examined the microbial “fingerprint” of feta PDO cheese, revealing that LAB, non-starter LAB, and yeasts are the predominant microorganisms [8].

Feta requires a ripening period of at least 2 months after which it can be released into the market either in brine or vacuum packed. A common consumer practice is to purchase a desired amount of feta from a larger brine-soaked package (often from a barrel) in the supermarket and then store the product in wrapping paper under aerobic conditions in the home refrigerator until consumption. However, this practice can lead to an increase of other non-essential microorganisms, such as yeasts in feta [8,9]. Yeast overgrowth can lead to significant defects [5,14]. Other microorganisms that can cause a problem in feta during storage are common foodborne pathogens i.e., *Listeria monocytogenes*, *Escherichia coli* O157:H7, *Yersinia enterocolitica*, *Salmonella* spp., and *Staphylococcus aureus*, that can be present in white brine cheeses due to bad hygiene conditions [5,8]. Among these microorganisms, *L. monocytogenes* can be a serious concern since it has been linked to fatalities in several cases involving the consumption of a variety of soft and semi-hard cheeses [15]. *L. monocytogenes* presence in dairy products is indicative of inadequate milk pasteurization [16] or post-pasteurization contamination because of insignificant hygiene conditions during cheesemaking [17]. Until now, *L. monocytogenes* presence in feta cheese was confirmed through shotgun metagenomics [8] and by the Rapid Alert System for Food and Feed (RASFF) since its presence was detected in an exported feta cheese product [18].

Biopreservation utilizes beneficial microorganisms and/or their metabolites to extend the shelf life and ensure food safety. Such microorganisms are LAB, which are commonly used as commercial starter cultures in the dairy industry [19], with many studies reporting that LAB strains produce antimicrobial substances and inherently help preserve products [20,21]. However, many studies have shown that NSLAB also could extend the shelf life of a variety of foods [22,23,24], as well as increase product safety levels [25,26]. LAB are generally recognized as safe (GRAS) [27], and many of them have been studied as biopreservatives because they produce organic acids or bacteriocins that may have antimicrobial action [24]. Additionally, the use of LAB cell-free supernatants (CFS) may be an alternative solution in food biopreservation, as they can exhibit antimicrobial properties [23] such as beef [28], chicken [29], shrimps [30], vegetables [31,32], and cheeses [33].

Controlling microbial populations at every stage of processing is crucial for the dairy industry. However, current microbiological analyses are expensive, require advanced equipment and highly skilled personnel, and the acquired results are often time-consuming and contentious. Moreover, these tests are usually destructive to the products being tested, limiting their use in real time or continuous monitoring scenarios [34]. These limitations, coupled with rapid technological advancements, have prompted the quest for novel methodologies within the dairy industry, such as the use of sensors. Spectroscopy-based sensors integrated with machine-learning techniques and their ability to be used online have been investigated for their capability to rapidly evaluate the microbiological quality and, by extension, the shelf life of various food products [35,36].

Based on the above, the objective of this study was to investigate the bioprotective effect of *Lactiplantibacillus pentosus* L33 and *Lactiplantibacillus plantarum* L125 free cells, as well as supernatants on feta cheese quality and the fate of *Listeria monocytogenes* during storage in air, in brine, and in vacuum at 4 and 10 °C using a comprehensive approach of analyses. The criteria used for the selection of these strains were that they were originally isolated from traditional fermented products presenting biofunctional properties [37]. In this context, *L. pentosus* L33 is a good probiotic candidate due to its tolerance to gastrointestinal tract conditions, susceptibility to common antibiotics, and *γ*-hemolytic activity [38], while *L. plantarum* L125 exerts anti-proliferative, anti-clonogenic, and anti-migration activity against the human colon adenocarcinoma cell line, HT-29 [39]. In addition, the study explored the feasibility of Fourier-transform infrared spectroscopy (FTIR) and multispectral imaging (MSI) to evaluate the microbiological quality of feta.

## 2. Materials and Methods

### 2.1. Experimental Design

Feta cheese was inoculated with *Listeria monocytogenes* cocktail strains and then treated with a co-culture of *Lactiplantibacillus pentosus* L33 and *Lactiplantibacillus plantarum* L125. These LAB strains, or their supernatants, were applied either directly or after being entrapped in Na-alginate edible films to study their effect as bioprotective cultures during storage under different packaging systems. Samples were subjected to microbiological, pH, a_w_, sensory, image, and spectroscopic analyses.

### 2.2. Microbial Cultures Preparation

The strains that were used in the current study were the following: *Lactiplantibacillus pentosus* strain L33 (previously isolated from fermented sausages) [37,38], *Lactiplantibacillus plantarum* strain L125 (previously isolated from fermented sausages) [37,39], and *L. monocytogenes* FMCC-B127 and FMCC-B133 (previously isolated from chicken salad and soft cheese, respectively). LAB strains belong to the culture collection of the Institute of Technology of Agricultural Products, Hellenic Agricultural Organization-DIMITRA, whereas FMCC strains belong to the culture collection of the Agricultural University of Athens, Greece. All strains were revived from pure cultures supplemented with 20% glycerol that were stored at −80 °C. *L. monocytogenes* strains (monocultures) and incubated overnight at 37 °C in 10 mL of Tryptone Soy Broth (TSB, NCM0019A, Neogen, MO, USA). A subculture of the pathogenic strains was inoculated in 10 mL of fresh TSB and incubated for 18 h at 37 °C. Centrifugation (6000× rpm, 10 min, 4 °C) of the overnight cultures followed, and the acquired pellet was washed twice with sterile ¼ strength Ringer’s solution (NCM0191K, Neogen), where the final pellet was resuspended in Ringer’s solution. To prepare the inoculant, the biomass of the two strains was mixed in equal volumes, and, accordingly, decimal dilutions were prepared to inoculate the cheese samples with an initial population level of approximately 3 log CFU/g. To confirm the inoculum size of *L. monocytogenes*, the inoculants were spread on *Listeria* Palcam Agar (Ref. 4016042, Biolife, Italiana S.r.l, Milano, Italy) containing Palcam selective supplement (*Listeria* Palcam Antimicrobic Supplement, Ref. 4240042, Biolife) and incubated at 37 °C for 48 h.

For LAB strains, the pure cultures were inoculated in 10 mL of de Man, Rogosa, and Sharpe (MRS) broths (MRS broth, 4017292, Biolife, Milano, Italy) and incubated overnight at 30 °C. A subculture of each strain was prepared in 10 mL of full fat (3.6%) UHT milk (FrieslandCampina Hellas S.A.—NOYNOY, Athens, Greece) obtained from a local retail market and incubated for 24 h at 30 °C. The cells were then harvested by centrifugation (6000 × rpm, 10 min, 4 °C), and the supernatant was collected under aseptic conditions for use in cheese inoculation. The pellet of the microorganism was also collected and subsequently washed with Ringer, as previously described. The pellet was then diluted in the appropriate amount of UHT milk to achieve a population level of 5 log CFU/g for cheese inoculation. To verify the inoculum, the inoculants were poured plated on MRS ISO agar (NCM0190, Neogen) and incubated at 30 °C for 48–72 h.

### 2.3. Na-Alginate Edible Film Preparation

The preparation of the Na–alginate edible films was performed as described in Pavli et al. [40]. In brief, 2% of Na-alginate edible films were prepared, using the cells or the supernatant of *Lactiplantibacillus pentosus* L33 and *Lactiplantibacillus plantarum* L125 strains. For the case of the cells, the pellet that was acquired by the centrifugation of the LAB strains, was mixed in equal volumes and added with agitation into the forming solution of Na-alginates, and the normal procedure was then followed. In the case where the supernatant of the LAB strains was used to prepare the edible films, the supernatant that was collected by centrifugation was added to the forming solution, with respect to the final volume of the forming solution (2%). Squared Petri dishes were used as matrix to form the edible films and approximately 20 g of Na-alginate solution (containing either the cells or their supernatant) were placed on the Petri dishes and allowed to dry for 12 h inside a laminar flow cabinet at ambient temperature. To detach the square films (ca. 0.5 g) from the Petri dishes, 20 mL aliquots of 2% *w*/*v* CaCl_2_ were added for 1 min, and then the films were dried and stored at 4 °C until use.

### 2.4. Inoculation of Feta Cheese Samples and Packaging of the Cheeses

Low-fat (5%) feta cheese from our industrial partner (Family Farm, Almyros, Volos, Greece) was transported to the laboratory in packages of 10 kg in 4% brine. The feta cheese that was used in the experiment has been ripened for 90 days and was sent to the laboratory before being released in the market, with a shelf life of 90 days in plastic containers.

Firstly, feta was cut into pieces of 150 g, and different scenarios of packaging and contamination were followed. In detail, feta (in portions of 150 g) was transferred inside a laminar flow cabinet and inoculation with *L. monocytogenes* strains followed by spraying of the pathogen on each cheese slice (150 μL of a 6 log CFU/mL dilution) to result in a population of 3 log CFU/g in the final product. Then, the slices (except the cases containing the Na-alginate edible films, see below) were sprayed with (i) 150 μL of UHT milk (to serve as the control, C samples), (ii) the supernatant (S samples) of LAB strains by spraying 150 μL of supernatant (that was acquired after overnight incubation of the strains in the UHT milk and was separated from the pellet by centrifugation; see Section 2.2) to cheese slices, and (iii) the cells (F samples) of LAB strains that have been diluted in UHT milk by using 150 μL of a 8 log CFU/mL culture of LAB strains to result in a population of 5 log CFU/g in the final product. Then, the slices were left for 5–10 min to absorb the cultures or the supernatants and were cut in pieces of 30 g to be packaged (a) under vacuum packaging by using sterile pouches using a HenkoVac 1900 machine (Howden Food Equipment B.V., Hertogenbosch, The Netherlands), (b) in 4% of fresh brine, and (c) under aerobic packaging. All of the aforementioned cases (UHT milk, cells, or supernatant of the LAB strains) were also prepared without the addition of the pathogen to the cheese.

For the cases of the slices that were wrapped with the Na-alginate edible films containing the pellet (FF samples) or the supernatant (SF samples) of the LAB strains, cheese samples were sprayed with the pathogen, wrapped with edible films, and then stored under vacuum packaging (as described above). Control samples (C) for this case were the same as control samples of vacuum conditions.

All samples of all cases were then stored at 4 °C and 10 °C in high-precision incubators (MIR153, Sanyo Electric Co., Osaka, Japan) until spoilage. A summarized description of the different cases and treatments is presented in Supplementary Table S1.

### 2.5. Microbiological Analysis

Microbial analyses of the feta cheese samples were performed during storage of the cheeses up until Day 90, depending on the different treatments of the samples and the different storage temperatures or packaging. To evaluate the number of viable cells, duplicate samples of each treatment were weighted (10 g) aseptically in sterile stomacher bags (BagLight^®^, INTERSCIENCE, Saint Nom la Bretêche, France) and sterilized ¼ Ringer’s (NCM0191K, Neogen) solution (90 mL) was added, and then samples were homogenized (60 s) at room temperature using a Stomacher (Lab Blender 400, Seward Medical, London, UK). Then, serial decimal dilutions were prepared by using ¼ strength Ringer’s solution and, accordingly, 0.1 or 1 mL of the appropriate dilutions were spread or poured on the following non-selective and selective agar media: Plate Count Agar (Tryptic Glucose Yeast Agar PCA, Ref. 4021452, Biolife, Italiana S.r.l, Milano, Italy) for the enumeration of Total Aerobic Viable Counts (TVC), incubated at 30 °C for 48–72 h; MRS ISO Agar (Neogen) overlaid with the same medium for the enumeration of mesophilic lactobacilli, incubated at 30 °C for 48–72 h; M17 Agar (Ref: 401719S2, Biolife, Italiana S.r.I. Milano, Italy) overlaid with the same medium, for the enumeration of lactic streptococci, incubated at 37 °C for 18–24 h; Violet Red Bile Glucose Agar (VRBGA, Ref. 4021862, Biolife) for the enumeration of *Enterobacteriaceae* counts overlaid with the same medium and incubated at 37 °C for 18–24 h; Rose Bengal Chloramphenicol Agar (RBC; LAB036, LAB M, Lancashire, UK) supplemented with selective supplement X009 (LAB M) for the enumeration of yeasts/molds, incubated at 25 °C for 48–72 h; *Listeria* Palcam Agar Base (Biolife) with Palcam selective supplement (Biolife) for the enumeration of *L. monocytogenes*, incubated at 37 °C for 24 and 48 h. Samples not inoculated with the pathogen were also examined for the absence of *L. monocytogenes*.

### 2.6. pH and a_w_ Measurements

The pH value of the cheese samples (inoculated and non-inoculated) was recorded using a digital pH meter Russell RL150 (Russell Inc, Cork, Ireland). At the end of the microbiological analyses, the glass electrode (Metrohm AG, Herisau, Switzerland) was immersed in the homogenized (stomacher bag, 1st decimal dilution) sample.

The water activity (a_w_) of each sample (inoculated and non-inoculated) was measured with a Novasina Thermoconstander RTD 33 water activity meter (Novasina AG, Zürich, Switzerland) at 25 °C.

### 2.7. Sensory Analysis

Sensory analysis was performed by a semi-trained panel consisting of nine laboratory staff members. All panelists provided their consent before participating in the study. Attributes that were evaluated were selected based on a previous study by Papadopoulou et al. [3]. In brief, panelists evaluated the appearance (white color and typical mechanical openings), the aroma (familiar i.e., buttery or mildly acidic, smell of the cheeses, rancid, and/or high acidic aroma of the samples), and the taste (in non-inoculated samples) (buttery, acid, sweet, salty, bitter, or rancid taste). A three-class evaluation scheme was employed in this experiment [41]. The first class (0–1, fresh) corresponded to fresh samples; the second class (1.5–2, marginal) to semi-fresh samples (2~accepted); and the third class (2.5–3, unacceptable) to spoiled samples. Samples that received negative evaluations on any of the assessed attributes from at least five panelists were deemed unacceptable. This specific day was then designated as the end of the sample’s shelf life.

### 2.8. FTIR-ATR Spectroscopy

FTIR analysis was performed using a ZnSe 45° HATR (Horizontal Attenuated Total Reflectance) crystal (PIKE Technologies, Madison, WI, USA) on an FTIR-6200 JASCO spectrometer (Jasco Corp., Tokyo, Japan). The spectrometer was equipped with a standard sample chamber, a triglycine sulfate (TGS) detector, and a Ge/KBr beamsplitter for the acquisition of spectral data. A representative volume of the cheese sample was cut into small pieces and then placed on the crystal surface and pressed by a gripper to ensure the best possible contact with the crystal. Crystal’s refractive index was 2.4 and a depth of penetration of 2.0 μm at 1000 cm^−1^. The spectra were obtained over the wavenumber range of 4000 to 400 cm^−1^ by accumulating 100 scans with a resolution of 4 cm^−1^ and a total integration time of 2 min, and the spectrometer was programmed with the Spectra Manager™ Code of Federal Regulations (CFR) software version 2 (Jasco Corp.). Background measurements and cleaning of the crystal surface are reported in detail in previous work of our laboratory [32]. The range of FTIR spectra that was used for further analysis was between 1800 to 900 cm^−1^. Two FTIR spectra replicates were collected from each sample of each trial (two different cheese batches). In total, 329 samples were acquired for feta samples stored in aerobic conditions, 364 for brine, 340 for vacuum, and 468 for vacuum with edible film (the edible films were removed before acquiring the spectra).

### 2.9. Image Acquisition and Segmentation

Multispectral image (MSI) analysis was conducted using the VideometerLab (benchtop-MSI) and VideometerLite (portable-MSI) instruments (Videometer A/S, Herlev, Denmark). Details regarding the VideometerLab instrument and the portable VideometerLite can be found elsewhere [32,42]. Feta cheese samples were placed on Petri dishes and then placed inside the Ulbricht sphere of the VideometerLab or manually transferred under the sphere of the portable VideometerLite, both of which have a top-mounted camera, and multispectral images of the samples were then acquired. For each sample, two image replicates were taken from both sides of the Petri dish. The VideometerLab system software (version 2.12.39) was used for image segmentation to select the region of interest (ROI) of the samples and remove non-relevant areas (e.g., sample background, Petri dish). Canonical discriminant analysis (CDA) was employed, resulting in segmented images. The outcome of this process was the calculated average value and standard deviation of the intensity of the pixels within the ROI at each wavelength. In total, 343 samples and 267 samples were acquired from VideometerLab and VideometerLite, respectively, for aerobic storage, 490 and 339 for brine storage, 491 and 347 for vacuum, and 484 and 292 for vacuum with edible film (the edible films were removed before acquiring the images).

### 2.10. Data Analysis

Partial least squares discriminant analysis (PLS-DA) was used to classify the feta samples of each storage condition as fresh, semi-fresh, and spoiled, according to sensory evaluation (total score 1–3), regardless of temperature and treatment, using data from the three examined sensors (FTIR, VideometerLab, and VideometerLite) based on a similar methodology [43]. For each dataset, stratified sampling was applied so that 70% of the data were used for training and 30% for testing the models. The models’ performance was evaluated based on three metrics: precision (%), sensitivity (%), and accuracy (%). Sensitivity (%) was calculated by the number of correctly classified samples in each class, divided by the total number of samples in the original class. Precision (%) was evaluated by the number of correctly classified samples in each quality class, divided by the number of samples correctly predicted by the model in each class. The total correct classification accuracy (%) was determined by the number of correct classifications across all classes divided by the total number of samples [44]. For data analytics, the software XLSTAT^®^ software (version 2023.3.1, Addinsoft, New York, NY, USA) [45] was used.

### 2.11. Statistical Analysis

The experiments on feta cheese samples were conducted using two independent batches (two different seasonal batches) with two replicates in each batch. Data were transformed to log CFU/g, and the average and standard deviation (SD) of log CFU/g were calculated for each sample point. Microbiological and pH results were analyzed for statistical significance (*p* < 0.05) with analysis of variance (ANOVA), and Duncan’s multiple range test was applied to determine the significant differences among the results. Calculations were performed using the XLSTAT^®^ software (version 2023.3.1, Addinsoft, New York, NY, USA) [45].

## 3. Results

### 3.1. Microbiological Analyses, pH, and Water Activity (a_w_)

**Figure 1:**
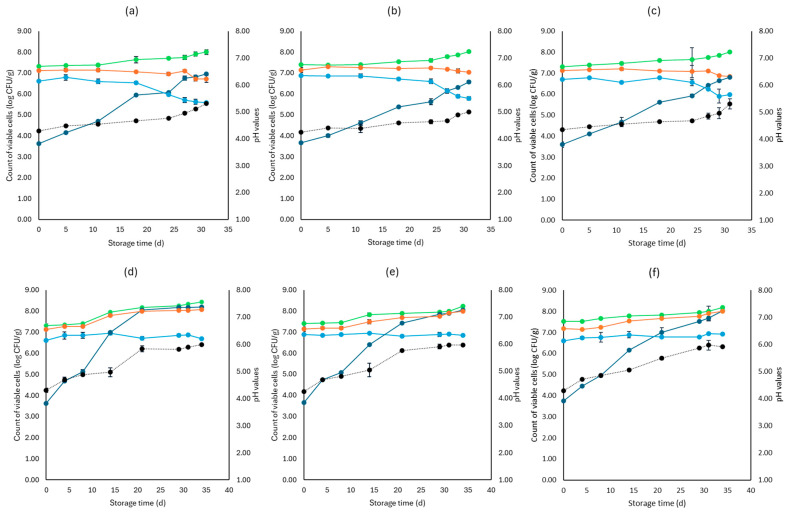
Population of the examined microorganisms and pH values in aerobic storage of non-inoculated feta cheese samples (mean values ± standard deviations) for (**a**): C samples stored at 4 °C, (**b**): F samples stored at 4 °C, (**c**): S samples stored at 4 °C, (**d**): C samples stored at 10 °C, (**e**): F samples stored at 10 °C, (**f**): S samples stored at 10 °C. (**•**) Total viable counts, (**•**) cocci/streptococci, (**•**) lactic acid bacteria, (**•**) yeasts and molds are represented by a continuous line (-). pH values (**•**) are indicated in the secondary axis and are represented with a dotted line (…). No statistically important differences were observed (*p* > 0.05).

**Figure 2:**
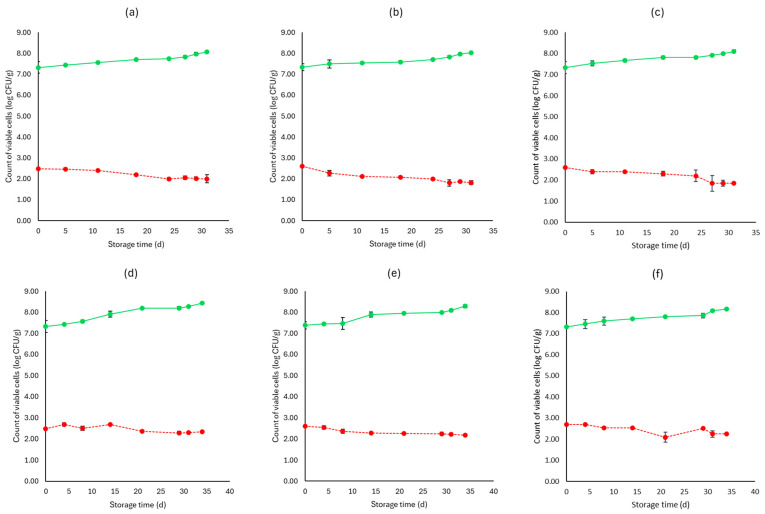
Population of total viable counts (TVC) and *Listeria monocytogenes* in aerobic storage of inoculated feta cheese samples (mean values ± standard deviations) for (**a**): C samples stored at 4 °C, (**b**): F samples stored at 4 °C, (**c**): S samples stored at 4 °C, (**d**): C samples stored at 10 °C, (**e**): F samples stored at 10 °C, (**f**): S samples stored at 10 °C. (**•**) TVC is represented by a continuous line (-), and *Listeria monocytogenes* (**•**) is represented in dashed lines (---). No statistically important differences were observed (*p* > 0.05).

**Figure 3:**
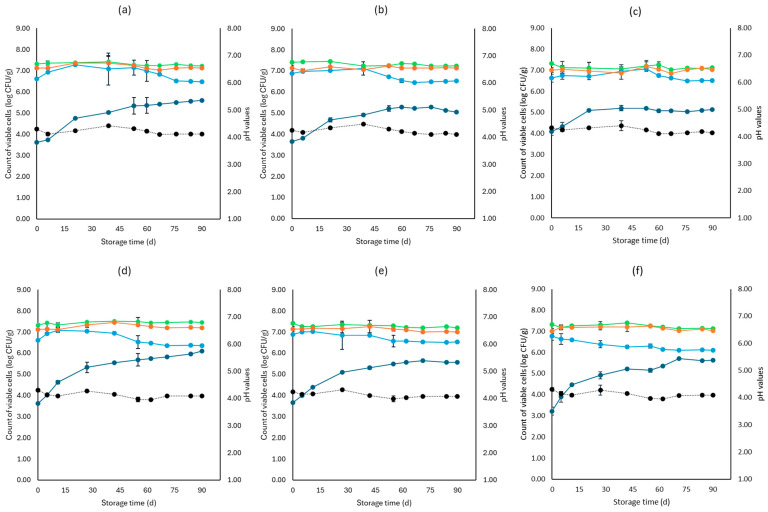
Population of the examined microorganisms and pH values in brine storage of non-inoculated Feta cheese samples (mean values ± standard deviations) for (**a**): C samples stored at 4 °C, (**b**): F samples stored at 4 °C, (**c**): S samples stored at 4 °C, (**d**): C samples stored at 10 °C, (**e**): F samples stored at 10 °C, (**f**): S samples stored at 10 °C. (**•**) Total viable counts, (**•**) cocci/streptococci, (**•**) lactic acid bacteria, (**•**) yeasts and molds are represented by a continuous line (-). pH values (**•**) are indicated in the secondary axis and are represented with a dotted line (…). No statistically important differences were observed (*p* > 0.05), except from TVC between C and both F and S samples and cocci/streptococci at 10 °C between C and S samples.

**Figure 4:**
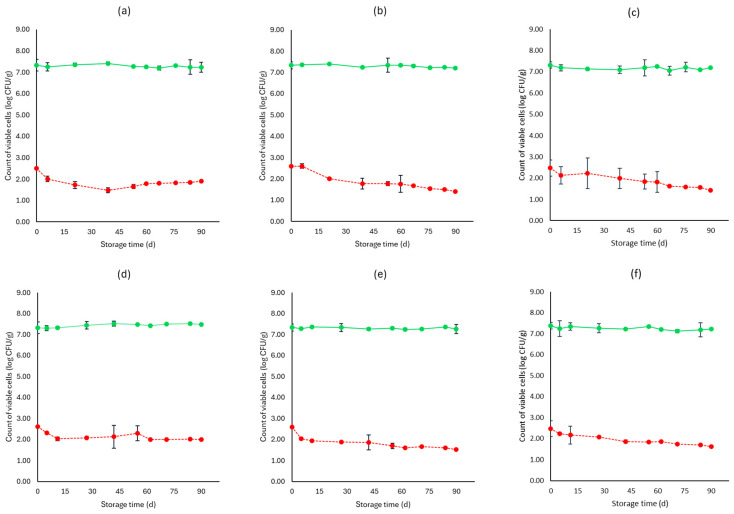
Population of total viable counts (TVC) and *Listeria monocytogenes* in brine storage of inoculated feta cheese samples (mean values ± standard deviations) for (**a**): C samples stored at 4 °C, (**b**): F samples stored at 4 °C, (**c**): S samples stored at 4 °C, (**d**): C samples stored at 10 °C, (**e**): F samples stored at 10 °C, (**f**): S samples stored at 10 °C. (**•**) TVC is represented by a continuous line (-) and *Listeria monocytogenes* (**•**) is represented in dashed lines (---). Statistically important differences (*p* < 0.05) were observed for TVC between C and both F and S samples.

**Figure 5:**
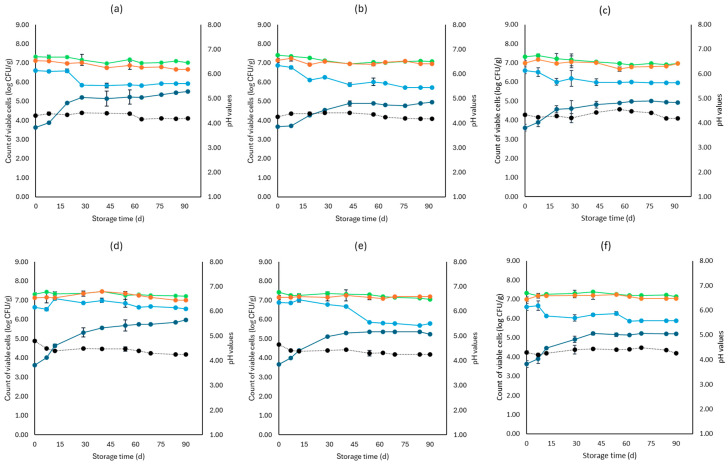
Population of the examined microorganisms and pH values in vacuum storage of non-inoculated feta cheese samples (mean values ± standard deviations) for (**a**): C samples stored at 4 °C, (**b**): F samples stored at 4 °C, (**c**): S samples stored at 4 °C, (**d**): C samples stored at 10 °C, (**e**): F samples stored at 10 °C, (**f**): S samples stored at 10 °C. (**•**) Total viable counts, (**•**) cocci/streptococci, (**•**) lactic acid bacteria, (**•**) yeasts and molds are represented by a continuous line (-). pH values (**•**) are indicated in the secondary axis and are represented with a dotted line (…). No statistically important differences were observed (*p* > 0.05) except from cocci/streptococci at 10 °C.

**Figure 6:**
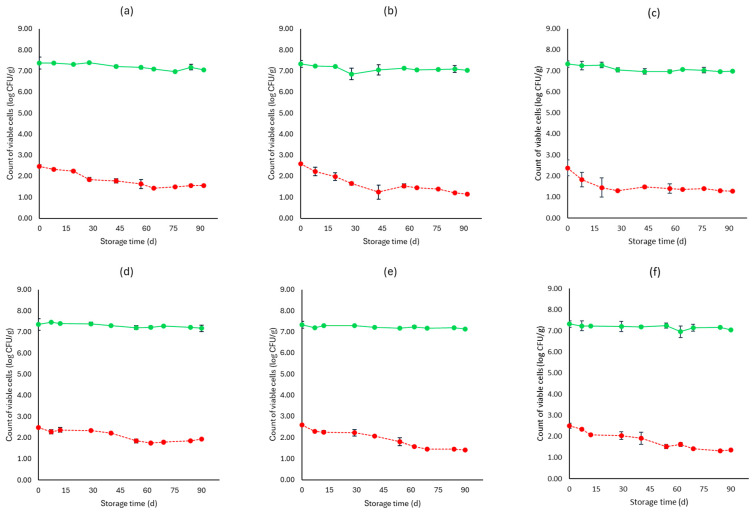
Population of total viable counts (TVC) and *Listeria monocytogenes* in vacuum storage of inoculated feta cheese samples (mean values ± standard deviations) for (**a**): C samples stored at 4 °C, (**b**): F samples stored at 4 °C, (**c**): S samples stored at 4 °C, (**d**): C samples stored at 10 °C, (**e**): F samples stored at 10 °C, (**f**): S samples stored at 10 °C. (**•**) TVC is represented by a continuous line (-) and *Listeria monocytogenes* (**•**) is represented in dashed lines (---). Statistically important differences (*p* < 0.05) were observed for *L. monocytogenes* between C and S samples.

**Figure 7:**
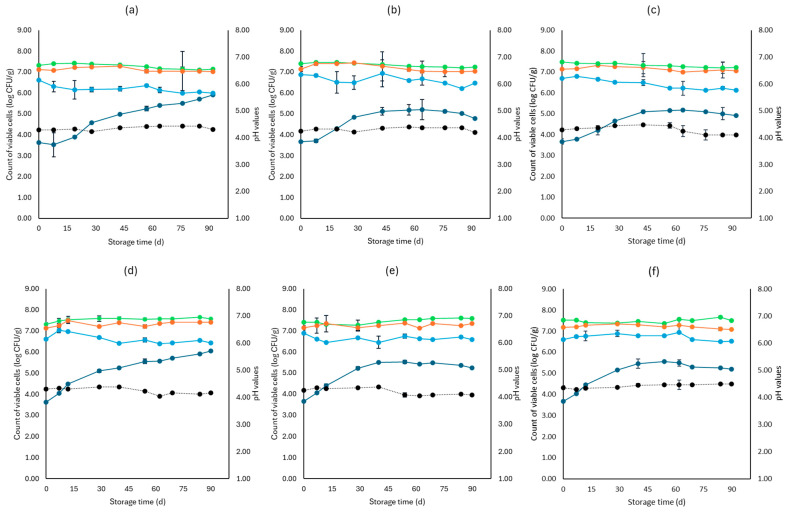
Population of the examined microorganisms and pH values in vacuum storage of non-inoculated feta cheese samples with edible film (mean values ± standard deviations) for (**a**): C samples stored at 4 °C, (**b**): FF samples stored at 4 °C, (**c**): SF samples stored at 4 °C, (**d**): C samples stored at 10 °C, (**e**): FF samples stored at 10 °C, (**f**): SF samples stored at 10 °C. (**•**) Total viable counts, (**•**) cocci/streptococci, (**•**) lactic acid bacteria, (**•**) yeasts and molds are represented by a continuous line (-). pH values (**•**) are indicated in the secondary axis and are represented with a dotted line (…). No statistically important differences were observed (*p* > 0.05) except from cocci/streptococci at 4 °C for C and FF samples.

**Figure 8:**
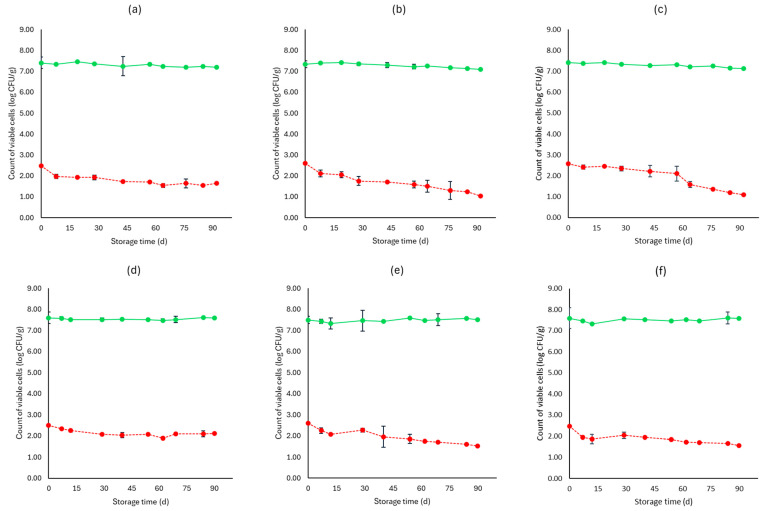
Population of total viable counts (TVC) and *Listeria monocytogenes* in vacuum storage of inoculated feta cheese samples with edible film (mean values ± standard deviations) for (**a**): C samples stored at 4 °C, (**b**): FF samples stored at 4 °C, (**c**): SF samples stored at 4 °C, (**d**): C samples stored at 10 °C, (**e**): FF samples stored at 10 °C, (**f**): SF samples stored at 10 °C. (**•**) TVC is represented by a continuous line (-) and *Listeria monocytogenes* (**•**) is represented in dashed lines (---). No statistically important differences were observed (*p* > 0.05).

Figure 1, Figure 2, Figure 3, Figure 4, Figure 5, Figure 6, Figure 7 and Figure 8 present the population of the examined microorganisms and the pH values of feta cheese samples under different storage conditions: aerobic, brine, vacuum, and vacuum with edible films. The initial TVC was 7.42 log CFU/g (±0.05), and the population of LAB and lactic cocci/streptococci was 7.17 log CFU/g (±0.13) and 6.72 log CFU/g (±0.10), respectively. For yeasts/molds, it was 3.63 log CFU/g (±0.02), while no *Enterobacteriaceae* were detected in the samples. The initial pH was 4.31 (±0.08) and a_w_ was 0.96 (±0.01), and the main microorganisms found in both storage temperatures (4 and 10 °C) were mesophilic LAB, followed by lactic cocci/streptococci and yeasts/molds, as seen in Figure 1, Figure 2, Figure 3, Figure 4, Figure 5, Figure 6, Figure 7 and Figure 8.

In aerobic packaging without the addition of the pathogen, TVC populations increased by the end of storage at both temperatures (4 and 10 °C) for all sample cases: control (C), cells (F), and supernatant (S), as seen in Figure 1. For C samples, the population of LAB at the end of storage at 4 and 10 °C was 6.70 log CFU/g (±0.32) and 8.07 log CFU/g (±0.03), respectively, while the population of lactic cocci/streptococci was 5.58 log CFU/g (±0.04) and 6.68 log CFU/g (±0.08), respectively. The yeast/mold population at the end of storage at 4 °C was 6.95 log CFU/g (±0.04), while for 10 °C, it reached higher values of 8.19 log CFU/g (±0.09). At the end of storage (4 and 10 °C) for F samples, population levels for LAB were 7.04 log CFU/g (±0.02) and 8.00 log CFU/g (±0.04), respectively, and for lactic cocci/streptococci 5.78 log CFU/g (±0.08) and 6.85 log CFU/g (±0.11), respectively, and were found similar to C samples (*p* > 0.05). The yeast/mold showed a slightly lower population in F samples compared to C samples during storage for both 4 and 10 °C (*p* > 0.05). Similar results to F samples were observed for S samples for both storage temperatures (*p* > 0.05). At the end of storage (4 and 10 °C), S samples had similar population levels of LAB (6.85 ± 0.07 log CFU/g, 8.01 ± 0.11 log CFU/g, respectively), and cocci/streptococci (5.98 ± 0.25 log CFU/g, 6.92 ± 0.02 log CFU/g, respectively) were compared to F and C samples (*p* > 0.05). During storage, samples were *Enterobacteriaceae* and *L. monocytogenes* free. For the cases of the cheeses inoculated with the pathogen, TVC values were similar to those of non-inoculated samples, as depicted in Figure 1 and Figure 2. Pathogen growth was stable across all examined cases (C, F, S), and no statistical differences were observed between the cases (*p* > 0.05). More specifically, at the end of storage (4 and 10 °C), F samples (1.81 ± 0.08 log CFU/g, 2.16 ± 0.01 log CFU/g, respectively) showed a slightly lower population compared to C (2.01 ± 0.02 log CFU/g, 2.34 ± 0.01 log CFU/g, respectively) and S (1.90 ± 0.02 log CFU/g, 2.21 ± 0.03 log CFU/g, respectively) samples. At the end of storage at both temperatures (4 and 10 °C), C samples had a pH value of 5.31 (±0.07) and 5.99 (±0.03), respectively, followed by S (5.30 ± 0.01 and 5.96 ± 0.02) and F (5.01 ± 0.05 and 5.91 ± 0.07) samples. The water activity (a_w_) across all treatments (C, F, S) remained consistent throughout storage at 0.96 ± 0.00 for 4 °C. At 10 °C, it slightly increased to 0.97 ± 0.00 by the end of storage. For inoculated samples, the population of the rest examined microbiota, pH, and a_w_ values were found similar to those of the cheeses without the pathogen and are not represented in Figure 2.

For samples that were stored in brine without the addition of the pathogen, TVC showed significant differences (*p* < 0.05) between C (7.22 ± 0.09 log CFU/g, 7.45 ± 0.01 log CFU/g, respectively) and both F (7.23 ± 0.07 log CFU/g, 7.21 ± 0.04 log CFU/g, respectively) and S (7.12 ± 0.07 log CFU/g, 7.13 ± 0.06 log CFU/g, respectively) samples at the end of storage of both temperatures. Furthermore, at 4 °C, the cocci/streptococci population of S samples showed a significant difference (*p* < 0.05) between the F and C samples during storage. Compared to aerobic storage, the growth of yeast/molds was kept at lower population levels at both 4 and 10 °C, as pointed out in Figure 1 and Figure 3. In more detail, population levels of yeasts and molds in C samples were 5.59 log CFU/g (±0.22) and 6.10 log CFU/g (±0.05), respectively, while in F samples, their population was 5.02 log CFU/g (±0.04) and 5.56 log CFU/g (±0.03), respectively, and at S samples, their populations were found 5.14 log CFU/g (±0.07) and 5.62 log CFU/g (±0.11), respectively, at the two storage temperatures. During storage, there was an absence of *Enterobacteriaceae* and *L. monocytogenes* in all samples. For the cases of the cheeses inoculated with the pathogen, like inoculated samples, TVC values were similar and showed a significant difference (*p* < 0.05) between the F and C samples. The growth of *L. monocytogenes* showed a decrease across storage in all cases, whereas, at the end of storage for 4 and 10 °C, its population in F (1.40 ± 0.03 log CFU/g, 1.52 ± 0.08 log CFU/g, respectively) and S samples (1.42 ± 0.05 log CFU/g, 1.62 ± 0.09 log CFU/g, respectively) had slightly lower population levels compared to C samples (1.91 ± 0.1 log CFU/g, 2.00 ± 0.06 log CFU/g, respectively). However, the changes observed were not significant (*p* > 0.05). The pH values were stable across storage, while at the end, the a_w_ was 0.97 ± 0.00 for all examined cases (C, F, S) and storage temperatures (4 and 10 °C). Also, the growth of the rest examined microbiota, pH, and a_w_ values, which were found similar between non-inoculated and inoculated cheese samples and are not depicted in the figure.

Regarding the vacuum-packaged feta cheese samples without the addition of the pathogen, the TVC and LAB populations remained relatively stable throughout storage at both temperatures and treatments, as observed in Figure 5. The cocci/streptococci population slightly decreased during storage at both 4 and 10 °C, as seen in Figure 5, and S samples exhibited a statistically significant difference compared to C samples (*p* < 0.05). In both storage temperatures (4 and 10 °C), the yeast/mold population was slightly lower at the end of storage at F (4.95 ± 0.04 log CFU/g, 5.23 ± 0.03 log CFU/g, respectively) and S (4.91 ± 0.03 log CFU/g, 5.19 ± 0.11 log CFU/g, respectively) samples, compared to C (5.55 ± 0.06 log CFU/g, 5.98 ± 0.05 log CFU/g, respectively) (*p* > 0.05). For all sample cases, *Enterobacteriaceae* and *L. monocytogenes* were absent during storage. For the cases of the cheeses inoculated with the pathogen, their population decreased during storage. More specifically, for both 4 and 10 °C, C samples (1.54 ± 0.21 log CFU/g, 1.92 ± 0.06 log CFU/g, respectively) at the end of storage had slightly higher population levels compared to F (1.15 ± 0.02 log CFU/g, 1.42 ± 0.02 log CFU/g, respectively) and S samples (1.28 ± 0.05 log CFU/g, 1.35 ± 0.05 log CFU/g, respectively). A statistically important difference between C and S samples was observed (*p* < 0.05), possibly indicating a slight effect of the supernatants of the LAB strains on the pathogen population. Similar to brine packaging, the pH values were stable across storage, as depicted in Figure 5, while the a_w_ did not differ between the examined cases (C, F, S) and storage temperatures.

For vacuum-stored, non-inoculated samples with the addition of edible films, microbial populations were similar to vacuum conditions for both 4 and 10 °C. The yeast/mold population for C samples showed a slight increase during storage for both temperatures (4 and 10 °C), with populations at the end of storage of 5.90 ± 0.02 log CFU/g and 6.05 ± 0.05 log CFU/g, respectively, while for FF (4.77 ± 0.02 log CFU/g, 5.23 ± 0.02 log CFU/g, respectively) and SF (4.91 ± 0.02 log CFU/g, 5.19 ± 0.02 log CFU/g, respectively) samples, populations were lower (*p* > 0.05). For samples inoculated with the pathogen, its population was slightly decreased at the end of storage (4 and 10 °C) for FF (1.03 ± 0.03 log CFU/g, 1.52 ± 0.02 log CFU/g, respectively) and SF (1.10 ± 0.02 log CFU/g, 1.56 ± 0.05 log CFU/g, respectively) samples, compared to C (1.65 ± 0.04 log CFU/g, 2.11 ± 0.06 log CFU/g, respectively) (*p* > 0.05). The pH values and a_w_ remained stable across storage. Also, inoculated samples had similar values for the rest of the examined microbiota, pH and a_w_.

### 3.2. Sensory Evaluation

**Figure 9:**
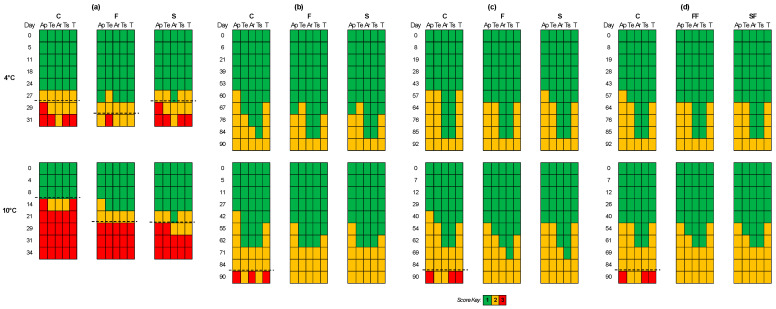
Sensory scores of aerobic (**a**), brine (**b**), vacuum (**c**), vacuum with edible films (**d**), storage of feta cheese samples during storage at 4 and 10 °C for appearance (Ap), texture (Te), aroma (Ar), taste (Ts), and total score (T). Dashed lines represent the end of shelf life.

Figure 9 displays the results of the sensory evaluation for the different cases and storage conditions examined (in non-inoculated samples). The shelf life of each examined storage condition was determined by the time point at which the corresponding sample was organoleptically rejected. Under aerobic storage at 4 °C, the shelf life of F samples was extended by 4 days compared to C and S samples, as seen in Figure 9, while at 10 °C, both F and S samples had an extended shelf life of 13 days compared to C samples. In brine storage for both temperatures, F and S samples had better scores for the examined attributes compared to C samples. More specifically, at the end of storage at 10 °C, C samples were rejected for their appearance, aroma, and total score compared to F and S samples that had lower score values. In vacuum storage at both temperatures, F samples had better score values at some time points compared to C and S samples. However, at the end of storage at 10 °C, the C samples were rejected for their appearance, taste, and total score compared to F and S samples. In vacuum storage with edible films, no significant differences in score values were observed between C, FF, and SF samples at storage for both 4 and 10 °C.

### 3.3. FTIR and MSI Spectral Data

**Figure 10:**
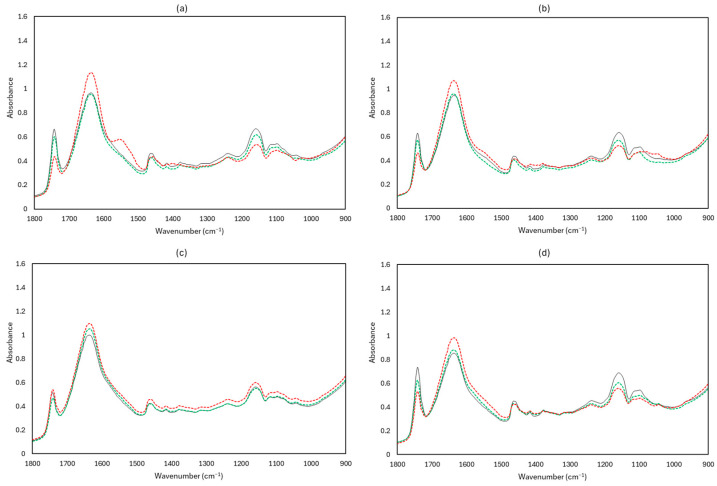
Raw Fourier transform infrared (FTIR) spectra, in the selected wavenumber range 1800–900 cm^−1^, corresponding to feta cheese samples stored under aerobic conditions (**a**), brine (**b**), vacuum packaging (**c**), and with edible film under vacuum packaging (**d**). Fresh samples (Day 0) are represented in black solid line (──), spoiled samples at 4 °C in green dashed line (----), and spoiled samples at 10 °C in red dashed line (----).

**Figure 11:**
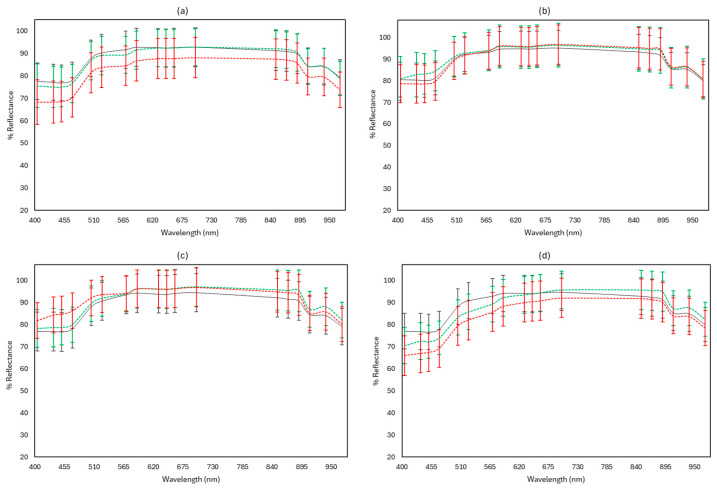
Indicative multispectral imaging (MSI) reflectance spectra (mean ± standard deviation) from the benchtop-MSI instrument, corresponding to feta cheese samples stored in aerobic conditions (**a**), brine (**b**), vacuum (**c**), and vacuum with edible film (**d**). Fresh samples (Day 0) are represented in black solid line (──), spoiled samples at 4 °C in green dashed line (----), and spoiled samples at 10 °C in red dashed line (----).

**Figure 12:**
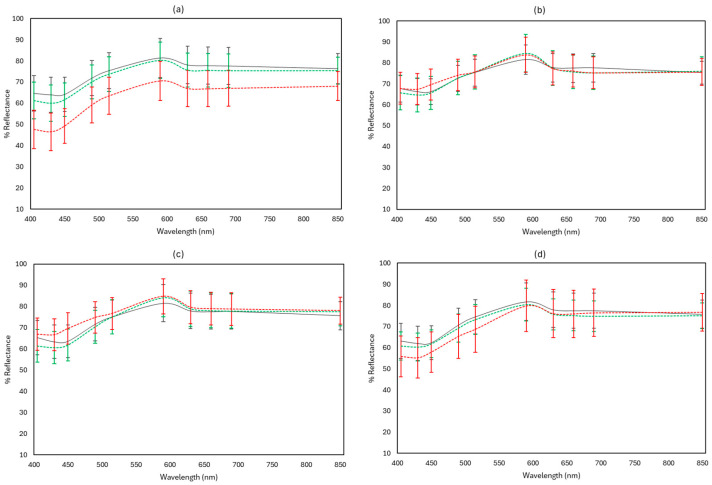
Indicative multispectral imaging (MSI) reflectance spectra (mean ± standard deviation) from the portable-MSI instrument, corresponding to feta cheese samples stored in aerobic conditions (**a**), brine (**b**), vacuum (**c**), and vacuum with edible film (**d**). Fresh samples (Day 0) are represented in black solid line (──), spoiled samples at 4 °C in green dashed line (----), and spoiled samples at 10 °C in red dashed line (----).

Figure 10, Figure 11 and Figure 12 display representative FTIR and MSI spectra of fresh (Day 0) and spoiled (end of storage) feta cheese samples under the different storage conditions examined at 4 and 10 °C, respectively.

Visual observation of the FTIR spectra (Figure 10) can provide some information on the differences in quality for the samples stored under different conditions. For instance, in all examined conditions, the peaks observed between 1680–1470 cm^−1^ showed an increase during storage at 10 °C. More specifically, in aerobically spoiled samples at 10 °C, the peaks at 1640 and 1550 cm^−1^ were more intense. The peaks around 1295–1000 cm^−1^ decreased at both 4 and 10 °C during storage, apart from vacuum conditions. According to the literature, peaks at 1640 cm^−1^ correspond to water (O-H) and amide I band (C = 0 stretch, C-N stretch, and N-H bend), while peaks at 1550 cm^−1^ could be assigned to the amide II band (N-H bend, C-N stretch). The minor peaks observed at 1460 cm^−1^, 1240 cm^−1^, and 1140 cm^−1^ could be attributed to lipids (CO esters). Also, some minor peaks with possible assignments were observed at 1150 cm^−1^ (fat C-O stretch, esters C–O–C, lactose carbohydrates C–O stretch, and –NH_2_ deformation), 1110 cm^−1^ (riboses C–O stretch and amines NH_2_ rock/twist), 1065 cm^−1^ (nucleic acids and phospholipids PO_2_ symmetric stretch/C-O stretch), and 1040 cm^−1^ (lactose and carbohydrates C–O stretch) [46,47,48,49,50,51,52].

As seen in Figure 11, for the majority of the 18 distinct wavelengths of the benchtop-MSI, the reflectance of spoiled samples at 10 °C was reduced in aerobic stored samples, as well as in samples wrapped with edible films and vacuum packed in comparison to fresh and spoiled samples held at 4 °C. However, no noticeable variations were observed in reflectance between fresh and spoiled samples for the samples stored in brine. Under vacuum conditions, spoiled samples at both 4 and 10 °C showed an overall increase in reflectance, compared to fresh samples. Spoiled samples at 10 °C exhibited a more intense increase in reflectance at the wavelength range 405–570 nm.

In the portable-MSI case, the spoiled samples stored in aerobic packaging at 10 °C showed a decrease in reflectance at all 10 wavelengths compared to fresh and spoiled samples at 4 °C, which was found similar to the benchtop-MSI spectra. Spoiled samples showed a decrease in reflectance in samples wrapped with edible film and stored under vacuum at 10 °C, as seen in Figure 12, in the wavelength range of 405–630 nm, in comparison to the other two cases. Except for spoiled samples at 10 °C, for which a visible increased reflectance at the range 405–505 nm can be observed, there are no major differences in reflectance between fresh and spoiled (4 and 10 °C) samples under vacuum packaging and brine storage.

### 3.4. Rapid Sensory Class Evaluation

Table 1, Table 2, Table 3 and Table 4 show the confusion matrices of the tested sets for the PLS-DA models applied to classify feta cheese samples in the predefined sensory cases for the storage conditions examined, using different spectroscopy-based sensors. For aerobic storage, the best accuracy score for the tested set was obtained from the benchtop-MSI data (72.79%), followed by FTIR (65.26) and portable-MSI (60.53%). More specifically, benchtop-MSI had the best balance between the misclassified classes on the safe side (i.e., fresh samples characterized as semi-fresh or spoiled, and semi-fresh characterized as spoiled) and those on the dangerous side (i.e., semi-fresh or spoiled samples characterized as fresh, and spoiled cheese characterized as semi-fresh). Safe-side misclassifications were 9.5% corresponding to 14 cases out of 147 samples and dangerous-side misclassifications were 17.7% (26 cases out of 147 samples). The sensitivity for spoiled samples was 73.81%, which is higher than the other two examined sensors, as seen in Table 1. Regarding the samples stored in brine, the PLS-DA models achieved the best accuracy scores for benchtop-MSI (89.12%) and portable-MSI (82.99%), in contrast to FTIR, which had a corresponding score of 76.19%. The benchtop-MSI was found to be the most reliable sensor, presenting the highest sensitivity in discriminating spoiled samples (100%) and with the lowest rates in both safe (6.5%, 14 cases out of 216 samples) and dangerous (7.4%, 16 cases out of 216 samples) misclassifications.

For samples packaged under vacuum, models from FTIR data showed the highest accuracy (84.31%), compared to benchtop-MSI (78.38%) and portable-MSI (71.92%). The PLS-DA models of samples wrapped with edible films showed similar accuracy scores for FTIR (69.50%), benchtop-MSI (76.03%), and portable-MSI (73.29%) instruments.

As displayed in Table 1, Table 2, Table 3 and Table 4, sensitivity for detecting spoiled cheese was relatively low in some cases, indicating challenges in identifying spoiled samples. However, precision for classifying spoiled samples remained high across all examined methods, suggesting that when a sample is classified as spoiled, it is done with a high degree of certainty. Across all storage conditions, FTIR demonstrated a consistent pattern of high sensitivity for semi-fresh samples, indicating a more reliable performance compared to benchtop- and portable-MSI. Most sensors performed well in detecting fresh samples, with FTIR and benchtop-MSI showing high sensitivity and precision across all storage conditions, while portable-MSI demonstrated adequate performance but with some limitations. The misclassifications among the three classes (Fresh/Semi-Fresh/Spoiled) could be attributed to the unbalanced sensory scores.

## 4. Discussion

The objective of this study was to investigate the bioprotective effect of *Lactiplantibacillus pentosus* L33 and *Lactiplantibacillus plantarum* L125 free cells and supernatants on the quality of feta cheese and the fate of *Listeria monocytogenes* during storage in air, in brine, and vacuum at 4 and 10 °C. It is well established that beneficial microorganisms or their metabolites may be used as biopreservatives to increase the shelf life and safety of foods. Such microorganisms are LAB, which are characterized as GRAS [27] and used as starter cultures in cheeses [8,19]. Many studies report that LAB strains produce antimicrobial substances to preserve products [20,21], such as organic acids and bacteriocins [24]. Organic acids are found in fermented dairy products because of bacterial growth during fermentation processes and normal biochemical metabolism [53]. The concentration of each organic acid can be influenced by starters, additional cultures, and non-starter lactic acid bacteria (NSLAB) present in cheeses [54,55]. Overall, organic acids present in cheeses play a crucial role in both flavor and preservation. Specifically, they lower the pH of dairy products, inhibiting the growth of harmful bacteria and spoilage microorganisms, thereby extending the product’s shelf life. Additionally, they contribute to the distinctive taste and texture of dairy products [56,57,58]. Furthermore, utilizing LAB cell-free supernatants (CFS) could serve as an alternative solution, as they possess notable antimicrobial properties [23,24], primarily due to the presence of several organic acids.

In the present study, the evolution of the microbiological characteristics of the cheese was thoroughly studied during its storage in different conditions simulating both good (commercial) practices (in brine or vacuum) and worst (aerobic preservation), which constituted a common practice at the domestic level of preservation. In addition, the microbiota of feta cheese was evaluated during storage at refrigerated temperatures and abuse.

The evolution of LAB during cheese preservation was similar at both storage temperatures, a result that has also been found in other studies [3]. During storage of the cheeses, the population of mesophilic LAB in all cases was stable and around 6–8 log CFU/g depending on the case, while yeasts/molds increased, especially during aerobic storage. However, mesophilic lactobacilli and lactic streptococci were found to be the dominant microbiota of the cheeses in all tests, as shown by the high population levels of the product during storage. Among the different treatments in the examined storage conditions, samples sprayed with supernatant (S) of the examined strains exhibited more significant differences between control (C) and samples sprayed with cells (F). More specifically, for vacuum storage, the decrease in pathogen population was found statistically significant in the case of S compared to C samples at both temperatures (4 and 10 °C), indicating a slight bioprotective role of the supernatant of the examined strains. At all storage conditions, except aerobic storage, the cocci/streptococci populations showed a greater decrease in population for S or SF samples, compared to C, F, or FF. The TVC populations were found significantly different in the case of samples stored in brine with the addition of the co-culture of LAB (cells and supernatant) compared to the C samples, indicating their possible bioprotective role in reducing the spoilage microbiota. The microbiological quality of the samples was found similar with other studied white-brined cheeses [3,59,60].

One of the key factors influencing the physicochemical and organoleptic characteristics of feta cheese is the microbial ecosystem present during its production, maturation, and storage of the product. The microbiology of feta cheese has been studied in some cases, and it is well-documented that various microorganisms are involved during its production [6,9]. Both NSLAB and enterococci diversity are a critical part of the microbiota of traditional cheeses in brine, such as feta [61,62]. Yeasts are often part of the secondary microbiota of these cheeses [63] and play an important role in the final stages of ripening [8,9]. It has been suggested that yeast contamination levels can negatively impact the organoleptic properties of Feta cheese and, consequently, its shelf life [5,14]. In the present study, especially for aerobic storage, the rapid growth of the yeasts also led to the quick rejection of the product. The treatment of the samples with the LAB in this study (F and S) led to an extension of the shelf life of the products during aerobic preservation. More specifically, at 4 °C, the shelf life of F samples was extended by 4 days compared to C and S samples, as shown in Figure 9, while at 10 °C, both F and S samples had a prolonged shelf life of 13 days compared to C samples. However, the shelf life of the products in aerobic storage was much shorter than samples stored in brine or under vacuum. This also constitutes the proper preservation practice of the Feta during transportation, marketing, and, most importantly, at home-level storage. In this respect, it must be emphasized that at the household level, the usual practice is to preserve Feta aerobically until consumption. Thus, the rapid growth of the yeasts will be favored resulting in the quick rejection of the product. In addition, the growth of yeasts that are usually acid and salt-tolerant metabolize the lactate produced by the starter lactic acid bacteria and generate NH_3_ from amino acids, causing a significant increase in the pH (>5). This deacidification process favors the growth of other undesirable microorganisms such as spoilage and/or pathogenic bacteria [5,64], a result that was also observed in the current study.

Although cheeses are considered safe food products, contamination with foodborne pathogens may occur at different stages of the food processing environment [65]. Contamination sources can include starter cultures, brine, packaging material, cheese tank or cheesecloth, cutting equipment, floors, and ripening areas, among others [17,65]. However, LAB cultures can enhance the safety of cheeses by inhibiting the growth of various pathogenic bacteria. In fact, numerous LAB strains have demonstrated antilisterial activity and many have been used as co-cultures in cheese fermentation to control *L. monocytogenes* [66]. In the present study, two strains of *L. monocytogenes* were inoculated into feta cheese for microbial risk assessment. Treatments with the LAB strains in the samples inoculated with the cocktail of the two pathogen strains during the aerobic, brine, and vacuum storage of the samples resulted in a slight inhibition of the pathogen compared to the control. Similar results have been noted in other studies, too. Prezzi et al. [67] studied the impact of *Lacticaseibacillus rhamnosus* GG on the growth of inoculated *L. monocytogenes* on the surface of Minas Frescal cheese, and results showed that the pathogen’s population was reduced. Morandi et al. [68] examined the influence of LAB strains on the growth of *L. monocytogenes* during the ripening of gorgonzola peels and observed growth inhibition after 50 days. Papadopoulou et al. [3] studied the behavior of *L. monocytogenes* during the ripening and storage of feta cheese both with and without the addition of *Lpb. plantarum* T571 as a supplemental culture and observed that the added LAB strain inactivated the pathogen more rapidly compared to the control samples.

A common practice to produce cheeses from pasteurized milk is the addition of commercial starter cultures, which standardize the process and can enhance their organoleptic properties. However, these cultures tend to produce a flat organoleptic profile in the products instead of a rich and distinct traditional flavor [19,69]. Therefore, the addition of selected native LAB strains to products as adjunctive cultures is receiving increasing attention [70,71,72]. These added microorganisms can produce intracellular enzymes during storage, primarily influencing the technological and organoleptic properties of cheeses [73,74]. Such microorganisms with special technological and functional properties are the LAB of the present study, which were not added during fermentation/ripening but sprayed onto the final product. From the results of the organoleptic evaluation of the study, it was observed that for the samples preserved in the brine for both temperatures, the F and S samples had better scores for the examined characteristics compared to the C samples. More specifically, at the end of storage at 10 °C, C samples were rejected for their appearance, aroma, and overall score compared to F and S samples that had lower score values. In vacuum storage at both temperatures, F samples were evaluated better than their counterparts C and S. Similar results have been pointed out by other researchers, such as González and Zarate [75], who examined a local Tenerife cheese made with a mixture of native strains (*Lc. lactis* subsp. *lactis* TF53, *Lactiplantibacillus plantarum* TF191, and *Leuconostoc mesenteroides* TF75) that displayed enhanced sensory characteristics compared to the control. Bancalari et al. [76] investigated the impact of a native *Lacticaseibacillus paracasei* strain as a co-culture to produce an Italian semi-hard cheese, resulting in an improved aroma profile, color, texture, and taste compared to the control. Additionally, many researchers have used indigenous LAB strains as co-cultures to produce feta cheese, noting an enhancement in organoleptic characteristics [3,41,55,77,78,79].

Industrial dairy products contain indigenous LAB strains that may be associated not only with desirable technological performance, as mentioned above, but also with health-related functions [41,80]. These microorganisms can be used as starters in food fermentations or as co-cultures with multifunctional characteristics [41,80]. The literature presents numerous reports of LAB isolation from various products, describing their potential as potential probiotic cultures and their further use in food [80,81,82,83,84]. In the present study, the LAB strains used have probiotic potential [37,38,39], which were used as co-cultures in fermented foods [40] and constituted the prevailing microbiota both at the end of fermentation and at the end of the lifetime of the examined products. Nevertheless, this specific object was not the purpose of the present study and was not further investigated.

FTIR spectroscopy and MSI analysis were applied as rapid tools to monitor biochemical changes in cheese during storage. Previous works involving different cheese products have evaluated the potential of FTIR spectroscopy to monitor cheese ripening [85,86,87], as well as to estimate the microbiological quality of cheeses [3]. MSI analysis has been used in the past to assess the microbiological quality of a variety of products [34]. However, there is limited, if any, information regarding MSI and the estimation of the microbiological quality of cheeses. In this study, for FTIR analysis, an increase in the peaks ranging between 1680–1470 cm^−1^ and a decrease in the range 1295–1000 cm^−1^ were observed for most of the different storage conditions in the case of spoiled samples. These findings agree with the study by Papadopoulou et al. [3] and could be attributed to the ripening of the cheese. Regarding the performance indicators of the models PLS-DA, they classified feta cheese samples as “fresh”, “semi-fresh”, and “spoiled” for the storage conditions examined, using different spectroscopy-based sensors. For aerobic storage, the best accuracy score for the test set was obtained from the benchtop-MSI data (72.79%), followed by FTIR (65.26) and portable-MSI (60.53%). In contrast, for brine conditions, the PLS-DA models achieved better accuracy scores of 89.12% and 82.99% for benchtop-MSI and portable-MSI, respectively, while for FTIR, a total accuracy score of 76.19% was observed. For vacuum storage, FTIR data had the highest accuracy (84.31%), compared to benchtop-MSI (78.38%) and portable-MSI (71.92%). The prediction results for FTIR agree with previous studies [43] for yoghurt sample classification according to sensory attributes using PLS-R. Across all storage conditions, the sensors performed well in detecting fresh samples, with FTIR and benchtop-MSI showing high sensitivity and precision. However, sensitivity for detecting spoiled samples was notably lower in several cases, which could lead to dangerous misclassifications where spoiled cheese is identified as fresh or semi-fresh, posing significant health risks. Therefore, high precision for identifying spoiled samples across all methods (100% in most cases) is particularly important for ensuring food safety, as it reduces the risk of false positives when spoilage is detected. All the above results of the study indicate that FTIR spectroscopy and MSI multispectral image analysis may be used in the future as important tools for the rapid detection of qualitative and structural changes during the storage of feta and, by extension, of other cheeses, too.

## 5. Conclusions

While the applied bioprotective LAB strains have demonstrated the potential to extend the shelf life of feta cheese and offer mild antimicrobial action against *L. monocytogenes* and spoilage microbiota, several areas warrant further investigation. Future research could focus on understanding the long-term stability and efficacy of these bioprotective strains under varying storage conditions, including different brine concentrations and packaging environments. Additionally, exploring the synergistic effects of combining bioprotective cultures with other natural preservatives could further enhance the safety and quality of feta cheese. The application of advanced, non-invasive sensor technologies like FTIR and MSI has shown promise in monitoring cheese quality, but further work is needed to refine these techniques for broader commercial use. Lastly, more studies are required to assess the consumer acceptance and sensory impact of these bioprotective interventions, ensuring that the extended shelf life does not compromise the traditional characteristics of feta cheese.

## Figures and Tables

**Table 1:** Confusion matrices and precision (%), sensitivity (%), and accuracy (%) scores for the test set of the PLS-DA models (fresh/semi-fresh/spoiled) for aerobic storage of feta cheese.

		Benchtop-MSI						Portable-MSI		
From/To	Fresh	Semi-Fresh	Spoiled	Precision (%)	Sensitivity (%)	Fresh	Semi-Fresh	Spoiled	Precision (%)	Sensitivity (%)
Fresh	46	14	0	75.41	76.67	28	6	0	82.35	82.35
Semi-fresh	15	30	0	54.55	66.67	5	10	0	29.41	66.67
Spoiled	0	11	31	100	73.81	1	18	8	100	29.63
**Accuracy (%)**	72.79					60.53				
		**FTIR**								
Fresh	43	22	0	93.49	66.15					
Semi-fresh	2	12	0	28.57	85.71					
Spoiled	1	8	7	100	43.75					
**Accuracy (%)**	65.26									

**Table 2:** Confusion matrices and precision (%), sensitivity (%), and accuracy (%) scores for the test set of the PLS-DA models (fresh/semi-fresh/spoiled) for brine storage of feta cheese.

		Benchtop-MSI						Portable-MSI		
From/To	Fresh	Semi-Fresh	Spoiled	Precision (%)	Sensitivity (%)	Fresh	Semi-Fresh	Spoiled	Precision (%)	Sensitivity (%)
Fresh	105	4	0	89.74	96.33	88	7	0	87.13	92.63
Semi-fresh	12	21	0	84.00	63.64	13	21	0	63.64	61.76
Spoiled	0	0	5	100	100	0	5	13	100	72.22
**Accuracy (%)**	89.12					82.99				
		**FTIR**								
Fresh	59	19	0	93.65	75.64					
Semi-fresh	4	20	0	48.78	83.33					
Spoiled	0	3	0	100	33.33					
**Accuracy (%)**	76.19									

**Table 3:** Confusion matrices and precision (%), sensitivity (%), and accuracy (%) scores for the test set of the PLS-DA models (fresh/semi-fresh/spoiled) for vacuum storage of feta cheese.

		Benchtop-MSI						Portable-MSI		
From/To	Fresh	Semi-Fresh	Spoiled	Precision (%)	Sensitivity (%)	Fresh	Semi-Fresh	Spoiled	Precision (%)	Sensitivity (%)
Fresh	105	9	0	98.13	92.11	75	11	0	78.13	82.21
Semi-fresh	2	4	0	11.76	66.67	21	12	0	37.50	36.36
Spoiled	0	21	7	100	25.00	0	9	18	100	66.67
**Accuracy (%)**	78.38					71.92				
		**FTIR**								
Fresh	51	10	0	94.44	83.61					
Semi-fresh	3	34	0	72.34	91.89					
Spoiled	0	3	1	100	25.0					
**Accuracy (%)**	84.31									

**Table 4:** Confusion matrices and precision (%), sensitivity (%), and accuracy (%) scores for the test set of the PLS-DA models (fresh/semi-fresh/spoiled) for vacuum storage of feta with edible film.

		Benchtop-MSI						Portable-MSI		
From/To	Fresh	Semi-Fresh	Spoiled	Precision (%)	Sensitivity (%)	Fresh	Semi-Fresh	Spoiled	Precision (%)	Sensitivity (%)
Fresh	55	9	0	77.46	85.94	43	10	0	64.18	81.13
Semi-fresh	16	51	0	72.86	76.12	24	53	0	77.94	68.83
Spoiled	0	10	5	100	33.33	0	5	11	100	68.75
**Accuracy (%)**	76.03					73.29				
		**FTIR**								
Fresh	46	25	0	79.31	64.79					
Semi-fresh	12	45	0	59.21	78.95					
Spoiled	0	6	7	100	53.85					
**Accuracy (%)**	69.50									

## Data Availability

The original contributions presented in the study are included in the article/supplementary material, further inquiries can be directed to the corresponding author.

## Supplementary Materials

The following supporting information can be downloaded at: https://www.mdpi.com/article/10.3390/microorganisms12091870/s1, Table S1: Different examined storage conditions and treatments of Feta cheese samples.
