# Supplementary material for: Effect of the Bioprotective Properties of Lactic Acid Bacteria Strains on Quality and Safety of Feta Cheese Stored under Different Conditions
Source: Microorganisms. 2024 Sep 10;12(9):1870. doi: 10.3390/microorganisms12091870 (PMC11434416; doi:10.3390/microorganisms12091870)
Supplement: Supplementary file 1 [file microorganisms-12-01870-s001.zip › microorganisms-3132960-supplementary.pdf]

Supplementary Table S1. Different examined treatments and storage conditions of Feta cheese samples.

Temperature	Storage condition	Treatment	Abbreviation	*L. monocytogenes*
4 °C / 10 °C	**Aerobic**	UHT milk (control)	C	Inoculated / Non-Inoculated
		Cells of L33+L125	F	
		Supernatant of L33+L125	S	
	**Brine**	UHT milk (control)	C	
		Cells of L33+L125	F	
		Supernatant of L33+L125	S	
	**Vacuum**	UHT milk (control)	C	
		Cells of L33+L125	F	
		Supernatant of L33+L125	S	
	**Vacuum + Edible Film**	UHT milk (control)/ No edible film	C	
		Edible film with free cells of L33+L125	FF	
		Edible film with supernatant of L33+L125	SF	
